# Wideband dynamic microwave frequency identification system using a low-power ultracompact silicon photonic chip

**DOI:** 10.1038/ncomms13004

**Published:** 2016-09-30

**Authors:** Maurizio Burla, Xu Wang, Ming Li, Lukas Chrostowski, José Azaña

**Affiliations:** 1 Institut National de la Recherche Scientifique—Énergie, Matériaux et Télécommunications (INRS-EMT), Varennes, Quebec, Canada J3X 1S2; 2 Department of Electrical and Computer Engineering, University of British Columbia, Vancouver, British Columbia, Canada V6T 1Z4; 3 State Key Laboratory on Integrated Optoelectronics, Institute of Semiconductors, Chinese Academy of Sciences, Beijing 100083, China

## Abstract

Photonic-based instantaneous frequency measurement (IFM) of unknown microwave signals offers improved flexibility and frequency range as compared with electronic solutions. However, no photonic platform has ever demonstrated the key capability to perform dynamic IFM, as required in real-world applications. In addition, all demonstrations to date employ bulky components or need high optical power for operation. Here we demonstrate an integrated photonic IFM system that can identify frequency-varying signals in a dynamic manner, without any need for fast measurement instrumentation. The system is based on a fully linear, ultracompact system based on a waveguide Bragg grating on silicon, only 65-μm long and operating up to ∼30 GHz with carrier power below 10 mW, significantly outperforming present technologies. These results open a solid path towards identification of dynamically changing signals over tens of GHz bandwidths using a practical, low-cost on-chip implementation for applications from broadband communications to biomedical, astronomy and more.

## Abstract (web summary)

Photonic-based instantaneous frequency measurement systems are usually bulky or require high optical power. Here, Burla *et al*. demonstrate an integrated measurement system that can identify frequency-varying signals in a dynamic fashion, without the need for fast measurement instrumentation.

## Graphical abstract

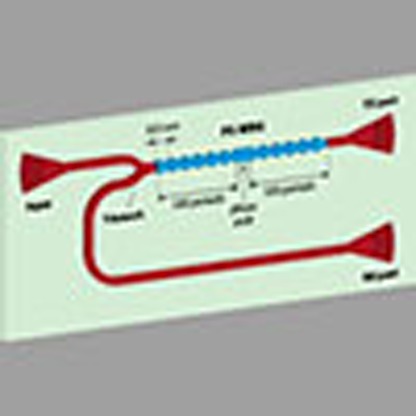

The capability to measure the frequency of unknown microwave and millimetre wave signals in a very fast manner is of primary importance for a wide range of applications in modern biomedical instrumentation^1^, electronic countermeasure (ECM), radar warning and electronic intelligence systems^2^,^3^,^4^. For this purpose, instantaneous frequency measurement (IFM) systems are employed. To further improve the bandwidth of operation and the reconfiguration capabilities of current electronic solutions, photonic-based IFM systems have been introduced in recent years^5^,^6^,^7^,^8^,^9^. Most demonstrations of photonic-based IFM solutions reported to date, however, are based on bulky and expensive off-the-shelf fibre optics components, therefore penalizing the overall system stability, compactness, measurement delay (latency) and unit cost. These factors are of critical importance in many applications, particularly for airborne ECM systems and biomedical devices. Recently, several demonstrations of integrated photonic IFM systems have been reported^10^,^11^,^12^,^13^,^14^, showing improved performance while providing a compact on-chip implementation. In most cases, however, these systems require high optical power (>100 mW) to enable nonlinear optical processing^10^,^11^,^15^, or are limited to bandwidths below 10 GHz (refs 12, 13), still below the frequency range of state-of-the-art electronic solutions (up to ≈18 GHz)^3^. Recently, Fandiño *et al*.^13^ proposed a very low-power solution (15 mW) based on ring-assisted Mach-Zehnder interferometers (RAMZI) on a III–V integration platform. This choice allows a relatively low-frequency error and, owing to the InP platform, shows great potential for monolithic integration of the system. Similarly to other solutions based on resonating structures, the drawback of this approach is that the RF bandwidth is limited to ∼10 GHz, still below state of the art electronic solutions, because of the finite free-spectral range (FSR) of the RAMZI filter used.

In this work, we propose and experimentally demonstrate an IFM design addressing, at the same time, both power and bandwidth issues of current photonic IFM solutions. The proposed system is based on a purely linear photonic solution, which uses a very compact waveguide Bragg grating (WBG) filter on-chip, 65-μm long only; it operates with a very low carrier power below 10 mW; and it provides an operating bandwidth above 30 GHz, only limited by the RF components available. To our knowledge, this represents the most compact and lowest optical power photonic-assisted IFM system proposed to date. The use of WBG solution constitutes a fundamental improvement as it eliminates the bandwidth limitation imposed by the finite FSR of previous solutions based on resonant filter structures^12^,^13^. In addition, owing to the extremely small waveguide propagation length, the system has the potential for an unprecedented latency reduction down to the tens of picoseconds level, several orders of magnitude lower than any previously reported photonic IFM system. Even the most recent reports are still based on techniques where tens of mm- or even cm-scale integrated devices are required^12^,^13^, together with hundred-milliwatts optical power levels. On the basis of this unique performance, we provide the first experimental demonstration of the capability of the proposed photonic IFM system for dynamic identification of instantaneous frequencies well above 10 GHz using instrumentation with detection/measurement bandwidths in the sub-GHz range and below.

## Results

### Device and operating principle

The operating principle of the proposed IFM system is based on a linear-optics frequency discriminator. The microwave frequency to be determined is mapped to a corresponding microwave power by means of the frequency-dependent response of a linear optical filter. Several solutions proposed to date employ not one, but two separate filter responses and define the frequency-discriminating function as the ratio of two powers, with the aim of increasing sensitivity and robustness^12^,^13^. This ratio is commonly referred to as the amplitude comparison function (ACF). In this work we employ a single, very simple and compact optical WBG filter on-chip, which we simultaneously operate in transmission and in reflection to provide two separate frequency responses used to define the ACF.

**Figure 1:**
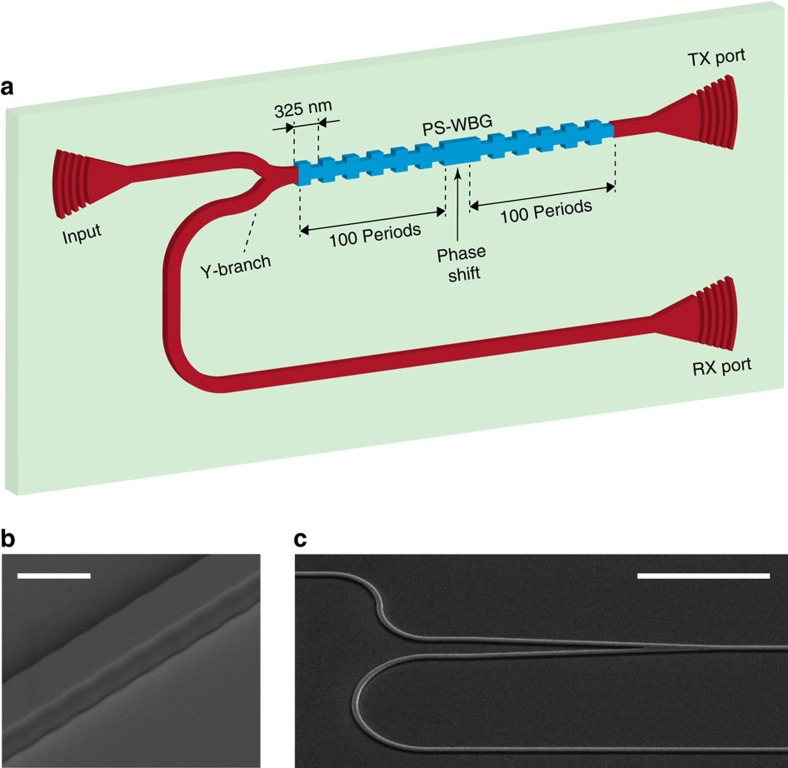
Silicon WBG. (**a**) Schematic of the silicon WBG (PS-WBG) employed as a linear-optics frequency discriminator. TX port, transmission port; RX port, reflection port. (**b**) Scanning electron microscope (SEM) image of the strip waveguide with sidewall corrugations (scale bar, 500 nm). (**c**) Y-branch used to access the reflection port of the PS-WBG (scale bar, 10 μm; images **b**,**c** from refs 16, 19).

The filter is physically realized with a phase-shifted WBG (PS-WBG) on a silicon-on-insulator (SOI) waveguide with a cross-section of 220 nm (thick) × 500 nm (wide). The chip was fabricated at IMEC using a complementary metal-oxide-semiconductor (CMOS)-compatible process^16^. A schematic of the device is shown in Fig. 1a. (See Methods for more details on the grating design and layout.)

A Y-branch, visible in Fig. 1c and connected as in Fig. 1a, is integrated on-chip to access both the transmission and reflection responses of the PS-WBG, avoiding the need for an off-chip optical circulator^17^,^18^. To the best or our knowledge, a WBG filter is here employed in an entirely different configuration, simultaneously using the device in transmission and in reflection, an approach that enables critical improvements in the accuracy and robustness of the measurement system. In fact, it is important to note that the use of the same optical filter to provide the two separate frequency responses defining the ACF translates into important advantages. In particular, this strategy allows us to keep an extremely simple layout for the filter while increasing the device robustness to variations of environmental conditions. For example, temperature fluctuations would create exactly the same frequency shift^19^ to both the transmission and reflection responses, thus being easier to compensate for in comparison with the case of using two physically separated filters.

**Figure 2:**
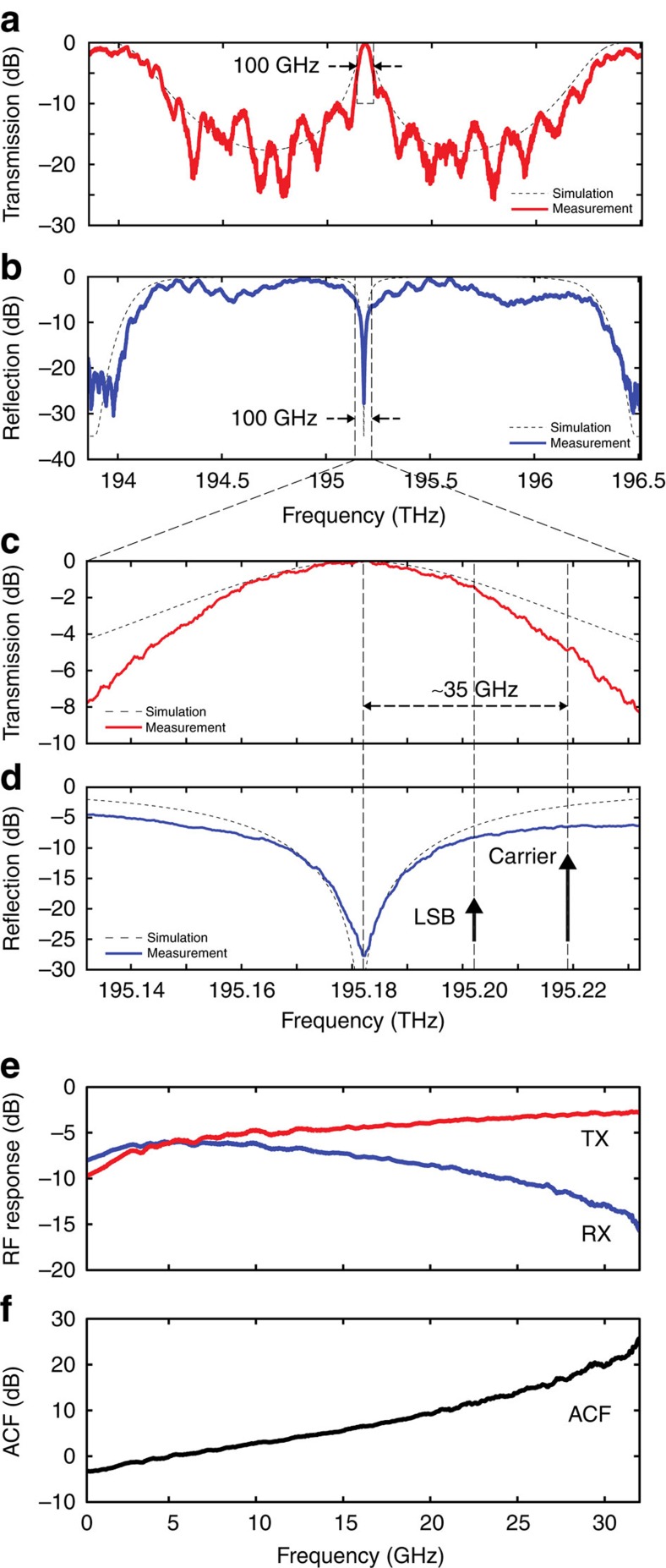
Optical and RF responses. Simulated (dashed line) and measured (solid line; **a**) linear optical transmission and (**b**) reflection spectral responses of the PS-WBG; (**c**,**d**) zoom with overlapped OSSB+C spectrum. The optical responses are normalized to the maximum. (**e**) RF response of TX and RX ports; (**f**) ACF, obtained from equation (1).

An optical vector network analyser (VNA, Luna Innovations OVA5000) was employed to characterize the optical transmission and reflection spectral transfer functions of the PS-WBG. The measured responses are shown in Fig. 2, normalized with respect to the maximum. The fibre-to-chip coupling loss is estimated ∼5 dB per facet. Approximately 4.5 dB is attributed to the grating coupler, and 0.5 dB to residual misalignment losses. Approximately 2 dB losses are attributed to the routing waveguides on-chip. The Y-junction adds 3.5 dB loss in the transmission path and 6.5 dB in the reflection path. The losses in the PS-WGB are considered negligible because of its ultrashort (<100 μm) length. Therefore, the overall fibre-to-fibre loss of the transmission and the reflection responses amount to ∼18 and 21 dB, respectively. As expected, these display opposite behaviour^20^: the transmission response shows a broad stopband with a single narrow peak in the middle, as in Fig. 2a,c, while the reflection response features a broad reflection band with a narrow notch, Fig. 2b,d. The transmission peak and the reflection notch occur at the same frequency; therefore, there is a frequency region where the slopes of the respective responses have opposite signs, Fig. 2c,d. These opposite responses with respect to frequency can be used to create a frequency discriminator for IFM, as will be described below. In Fig. 2a–d, the discrepancies between the simulated and measured responses are mainly because of the unwanted reflections at the fibre-to-chip coupling interfaces that create etalon fringes in the optical response. Nonetheless, experimental tests (Supplementary Note 1) show that these do not induce major perturbations in the ACF, even in presence of temperature changes (Supplementary Figs 1 and 2). Therefore, the proposed IFM system can be used at different operating temperatures. In this experiment, the device is temperature-stabilized at 21 °C during operation. In practical applications, the fibres could be glued to the chip to further reduce the effects of fluctuations of the etalon fringes.

We emphasize that the use of a WBG filter allows us to overcome the inherent RF bandwidth limitation of previous on-chip IFM demonstrations associated with the use of optical filters with a frequency-periodic response (for example, optical ring resonators^12^). In these solutions, the unambiguous range was always limited to half of the FSR or less^13^. The fact that the WBG does not have a frequency periodic response, instead, enables realization of IFM systems with theoretically unlimited RF bandwidth, practically constrained only by the operation (reflection) spectral bandwidth of the WBG filter, easily reaching the THz range. In fact, the bandwidth of the WBG notch, and associated operating RF bandwidth of the ACF, can be increased by simply using a shorter WBG. Simulations of PS-WBG responses with different lengths are reported in Supplementary Note 2. In addition, differently from the use of optical ring resonators^12^, the WBG suppression does not decrease when increasing the notch bandwidth by shortening the grating length: as the grating length is reduced, the notch spectral bandwidth is increased without modifying the WBG rejection peak, that is, the slope of the notch is correspondingly reduced (Supplementary Fig. 3). Therefore, the use of a WBG allows us to increase the unambiguous measurement range without reducing the dynamic range of the ACF.

### Experimental demonstration

**Figure 3:**
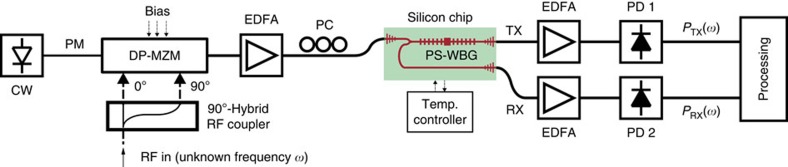
IFM system set-up. Schematic of the experimental set-up of the IFM system, including continuous-wave laser (CW), polarization maintaining fibre (PM), polarization controller (PC), dual-parallel Mach-Zehnder modulator (DP-MZM), erbium-doped fibre amplifier (EDFA), phase-shifted waveguide Bragg grating (PS-WBG) and photodetector (PD). RX, reflection port; TX, transmission port.

A schematic of the experimental set-up is shown in Fig. 3. The optical carrier is provided by a tunable laser (NetTest Tunics Plus) with +9 dBm continuous-wave optical power. The RF signal of unknown frequency *ω* enters a 90° RF hybrid coupler (ET Industries). The two 90° out-of-phase outputs are fed to the RF inputs of a dual-parallel Mach-Zehnder modulator (DP-MZM, Covega Mach-10 086) using coaxial cables of equal length. Properly biasing the modulator^21^ it is possible to create an optical single-sideband-modulated signal with full carrier (OSSB+C), where the upper sideband is suppressed and only the carrier and lower sideband are kept, as illustrated in Fig. 2d. A sideband ratio of 9.3 dB or higher is achieved over the complete bandwidth of operation by accurately biasing the DP-MZM (Supplementary Note 3 and Supplementary Fig. 4) At the DP-MZM output, the frequency separation between carrier and the remaining sideband is equal to the RF frequency *ω* to be determined. The OSSB+C modulated signal is amplified to compensate for the DP-MZM insertion loss and enters the optical chip input with an average power of ≈10 dBm (10 mW). It is important to note that this value is ∼10-fold lower than the optical power needed in on-chip IFM systems previously reported in literature (100 mW (ref. 12) or higher^10^,^11^,^15^). Fibre-to-chip coupling is realized using on-chip grating couplers^16^. Polarization controllers are placed before the DP-MZM and the optical chip to make sure the correct polarization is used and the power transfer is maximized. The optical carrier frequency is set at 1,535.66 nm (≈195.22 THz), ∼35 GHz apart from the transmission peak (and the reflection notch) of the PS-WBG, as shown in Fig. 2c,d. After it enters the chip, the modulated optical signal is partly transmitted and partly reflected by the grating, following two different paths, and outputs the chip from the transmission port (TX) and reflection port (RX), respectively. According to the responses in Fig. 2c,d, the optical sideband will experience an attenuation that decreases (respectively, increases) with frequency in the transmission (respectively, reflection) path as the RF frequency is increased. After amplification, the output signals from the TX and RX ports are detected using 45-GHz photodetectors (PDs, NewFocus 1014) with +3.5 dBm maximum optical input power. Erbium-doped fibre amplifiers (EDFA, Pritel Inc.) each with a noise figure of 8 dB are used to amplify the signals at the output of the transmission and reflection ports of the PS-WBG discriminator. Optical amplification is necessary in order to compensate for the losses due to fibre-to-chip and chip-to-fibre optical coupling. These amount to a total of ∼18 dB losses, which have been removed from Fig. 2 for clarity.

The average RF powers at the output of each PD, *P*_TX_(*ω*) and *P*_RX_(*ω*), are given by the power of the mixing products between the carrier and the lower sideband, after propagation through the transmission and reflection response of the PS-WBG, respectively. The functions |*s*_21,TX_| and |*s*_21,RX_|, in linear scale, indicate the amplitude (voltage) ratios between the transmission and reflection RF outputs, respectively, and the RF input. These functions have been measured using a VNA (Agilent E8364B) over the frequency range between 10 MHz and 32 GHz, and are displayed (in decibel) in Fig. 2e. As expected, they show opposite trends with frequency. The ACF of Fig. 2f is defined as the power ratio


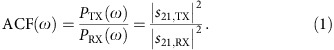


**Figure 4:**
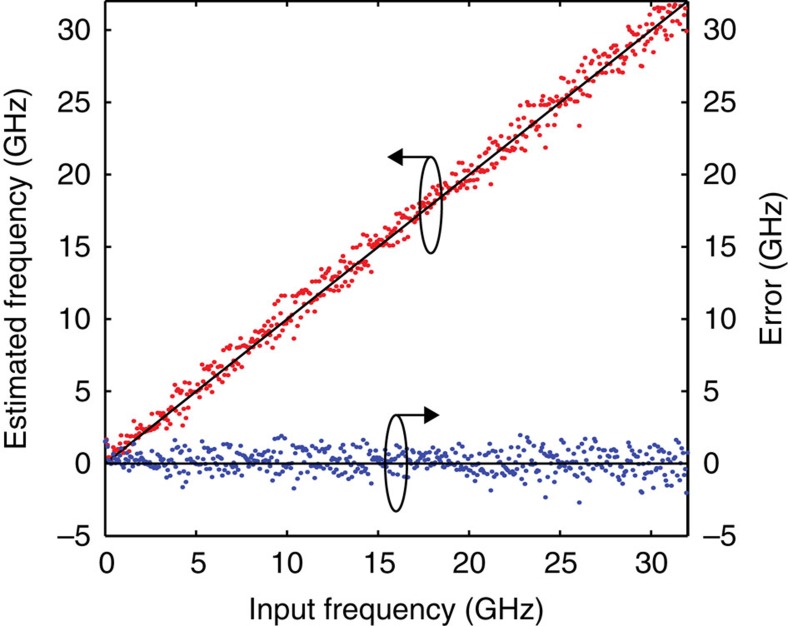
Frequency estimation. Estimated frequency (red dots) and corresponding error (blue dots).

The VNA was used to generate different RF test tones, with a constant power of −15 dBm. The corresponding output powers at the output of PD1 and PD2 can be measured at port 2 of the VNA. By inverting the ACF curve in Fig. 2f, the power ratio in equation (1) can be used to estimate the input frequency. The scatter plot in Fig. 4 shows the microwave frequency estimated using the IFM system versus the actual input frequency for each of the test tones, as well as the residual measurement error^22^. The figure shows that the proposed system is capable of estimating frequencies up to 32 GHz with a s.d. *σ*_RF_ of ∼755 MHz (root mean square error ≈773 MHz). The frequency uncertainty is attributed to the degradation of the noise figure of the photonic link, primarily because of the high fibre-to-chip coupling losses, and to the amplified spontaneous emission noise added by the EDFAs needed to compensate for these coupling losses. There is also an additional contribution from the thermal noise of the VNA employed for RF power measurements. See Supplementary Note 4 and Supplementary Figs 5 and 6 for a detailed analysis of the frequency estimation error.

### Dynamic frequency identification

**Figure 5:**
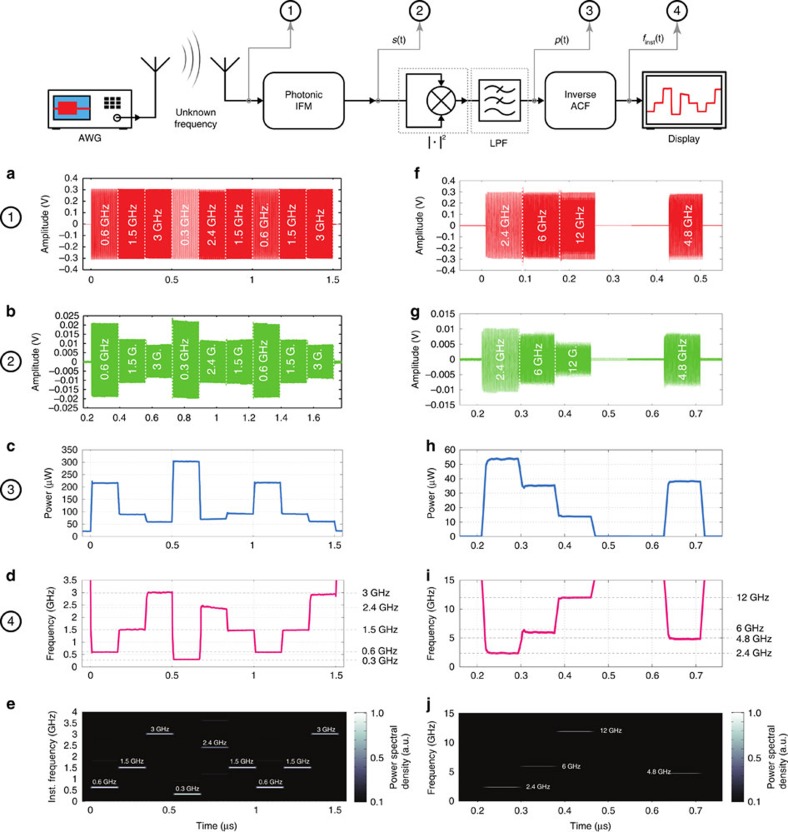
Experiment of dynamic frequency identification. (**a**) An RF signal with unknown frequency content enters the photonic IFM system in Fig. 3. (**b**) The time-domain signal at the IFM output is amplitude-coded according to the time-varying frequency content of the input signal. (**c**) The instantaneous power is extracted by self-mixing and low-pass filtering. (**d**) Using the inverse ACF, the RF frequency content is estimated in a dynamic manner. (**e**) The spectrogram of the frequency-hopping input sequence is shown for comparison. (**f**–**j**) Input sequence with frequency components at 2.4–6–12–4.8 GHz: (**f**) input waveform; (**g**) waveform processed by the IFM system, (**h**) average power extracted by mixing and low pass filtering, (**i**) estimated instantaneous frequency, (**j**) calculated spectrogram of the input sequence, shown for comparison to the estimated frequency in (**i**).

In this section, we experimentally demonstrate the capability of the proposed IFM design to perform dynamic microwave frequency identification. The scope of this test is to show that the system can accurately identify the temporal frequency profile of unknown, rapidly varying RF signals in a dynamic manner. A schematic of the experimental set-up and procedure is shown in Fig. 5.

A microwave signal with spectral content rapidly varying in time is generated and fed to the RF input of the photonic IFM system shown in Fig. 3. In a possible application scenario, this could represent an unknown RF signal of various different origins, for example, a biomedical signal, a radar pulse, an ECM jammer, and so on^1^,^2^. In our experiment, such a signal is chosen to be a frequency-hopping sequence, generated using an arbitrary waveform generator (AWG, Tektronix AWG7122C), as shown in Fig. 5. (Details on the programmed test sequences are reported in Methods.)

As visible in Fig. 5a, the amplitudes of the different frequency bursts have been carefully equalized in order to determine the actual capability of the IFM system to accurately discriminate the instantaneous frequency, without any additional reference. For this experiment, a PD with similar responsivity but higher power handling (Finisar BPDV2120R-VF-FA, capable of receiving +10 dBm of continuous-wave optical input power) has been employed in the set-up in Fig. 3 in place of the NewFocus 1014 that was previously used in the static IFM demonstrations. This is done to obtain a higher output photocurrent and compensate for the limited sensitivity of the real-time oscilloscope used in the tests. It is important to note that the real-time scope is used here only as a signal characterization tool, for example, for visualization, calibration and/or comparative purposes, and is not a part of the dynamic frequency identification system. Notice also that only one of such high-power PDs was available, and as a result, only one channel (the reflection channel, RX) of the IFM system could be employed in the dynamic tests. As a consequence, only the RF reflection response (RX), similar to the one in Fig. 2e, was employed as a frequency-discriminating function in place of the ACF in Fig. 2f. Therefore, the accuracy and sensitivity of the dynamic estimation can still be largely improved, especially at high RF frequencies, by simply employing another high-power PD connected to the transmit port of the PS-WBG, as in the set-up in Fig. 3, and using the complete ACF shown in Fig. 2f.

After processing through the IFM system, the sequence whose frequency is to be determined appears to be ‘amplitude-coded', as visible in Fig. 5b. In other words, differently from the input sequence, the frequency information is now mapped in the signal amplitude *s*(*t*) as well, as a consequence of the processing by the IFM discriminator. In order to extract the frequency information from the amplitude, without using any fast instrumentation, the average power (on a reference 1 Ω load) over a time interval Δ*T* of the signal *s*(*t*) originating from the IFM output is extracted using a self-mixing and low-pass filtering scheme:


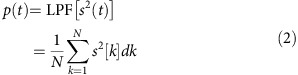


where *s*(*t*) indicates the (real) signal at the IFM output in Fig. 5b whose frequency is to be determined, *s*[k] is its sampled version and LPF[·] indicates the low-pass filtering operation. In this demonstration, the filtering was performed using a moving average filter as in equation (2) with *N*=400 samples. At the sampling rate of 40 GSa s^−1^ (sampling time of 25 ps) used in the experiment, this corresponds to an averaging time interval Δ*T*=10 ns. The resulting power *p*(*t*) (normalized to the resistance) is indicated by the time-varying signal shown in Fig. 5c. The value of the averaging time Δ*T* is chosen to be higher than the period of the lower-frequency component to be identified, in this case 0.3 GHz (period: ≈3.33 ns).

**Figure 6:**
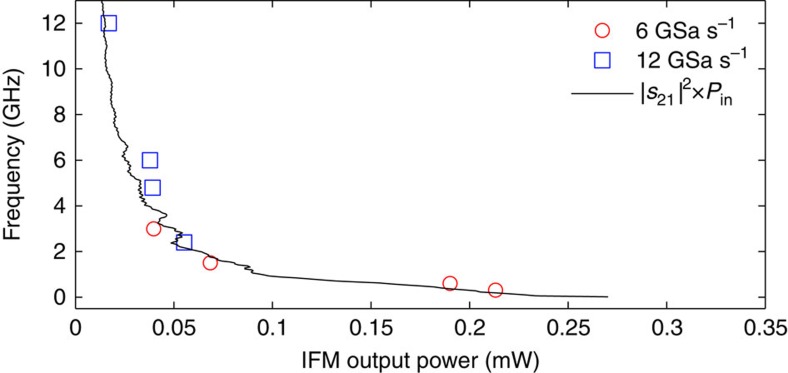
Inverse ACF employed in the dynamic IFM tests. The inverse ACF is used to estimate the frequency from the measured power. Red circles: calibration using 6 GSa s^−1^ input sequences; blue squares: calibration using 12 GSa s^−1^ input sequences; dashed line: magnitude squared of the transmission scattering parameter |*s*_21_|^2^ multiplied by the input RF power.

At this point, the inverse ACF shown in Fig. 6 is employed to map this average power into a frequency profile. As mentioned before, in this configuration, employing the high-power PD, the ACF is given by the RF reflection response only. This has been determined experimentally; in detail, the system has been calibrated before operation by feeding individual tones with the same amplitude of 0.3 V peak-to-peak at different microwave frequencies and recording the corresponding output amplitudes. The relationship between the output RF power and frequency was then inverted (Fig. 6) and employed as the inverse ACF in the system in Fig. 5. As expected, the obtained relationship coincides with the square magnitude of the transmission scattering parameter (|*s*_21_|^2^, measured with the VNA) multiplied by the input power *P*_RF,in_, see plot in Fig. 6. Residual deviations can be attributed to different EDFA settings and slightly different impedance-matching conditions during the spectral characterization and the dynamic experiments. The resulting instantaneous frequency profile is successfully extracted as shown in Fig. 5d.

## Discussion

A direct comparison of the result in Fig. 5d with the spectrogram of the input signal in Fig. 5e shows that frequency identification is performed in an accurate manner. Relatively low-frequency variations can be detected (300 MHz in this example) and the same frequency can be identified at different time slots in a highly reproducible manner. This is also shown in Fig. 5d, where the frequency step sequence 0.6–1.5–3 GHz was chosen to appear twice, at the beginning and at the end of each burst.

It is important to note that the 755-MHz s.d. value reported in the static system characterization is achieved when a single-shot identification is considered. In the practical dynamic frequency identification set-up shown in Fig. 5, a low-pass filtering is employed to extract the average power of the signal at the output of the PD. The low-pass filtering effect applied to the squared signal *s*^2^(*t*) is equivalent to a time-averaging of the same signal over a number of samples *N*. The time averaging reduces the noise variance and, therefore, increases the estimation accuracy compared with the static case. By choosing a larger value for *N*, the estimation error can be further reduced. There is a tradeoff between the time constant of the low-pass filter and the estimation error. We have provided a numerical comparison example in Supplementary Note 5, which shows that increasing the value of *N* from 40 to 400 increases the transient time between frequency bursts but also improves the frequency estimation accuracy (Supplementary Fig. 7). The system offers the flexibility to optimize the value of *N* based on the specific application requirements.

It is important to reiterate that, although the instantaneous frequency signal, *f*_inst_(*t*), contains frequency information in the tens of GHz range, it can be detected with a comparatively much lower speed electronic circuit, for example, a simple comparator with a bandwidth below a few hundreds of MHz in the presented examples. In particular, the circuit should simply be fast enough to detect the frequency ‘variations' of the incoming signal under test, and not its fast time-domain features and oscillations, including the microwave carrier oscillations, which themselves can reach or exceed tens of GHz frequencies. A detailed analysis of the transient response of the demonstrated IFM system is presented in the Supplementary Note 5. In spite of not being fully optimized, our IFM system is shown to be capable of accurately identifying nanosecond-scale dynamic transitions, matching or surpassing the performance requirements of state-of-the-art IFM systems for communications^4^, among others.

Further tests have been performed to demonstrate the capability to identify higher-frequency signals (Fig. 5f–j). A new sequence has been programmed using the same AWG, with frequency components at 2.4, 6, 12 and 4.8 GHz, including a 168-ns pause, as shown in Fig. 5f. Each burst has a duration of 84 ns. The PD output is displayed using the real-time oscilloscope and shown in Fig. 5g. The results in Fig. 5i–j show successful dynamic frequency identification up to 12 GHz, limited by the maximum frequency that could be generated with the available AWG (24 GSa s^−1^).

Finally, it should be also highlighted that the μm-scale dimensions of the used optical WBG filter should enable the implementation of practical photonic IFM systems with latency times as short as tens of picoseconds. Sub-100s-picosecond latency times can make a difference in important application scenarios, such as in the context of ECM, for example, jamming of hostile radars. To elaborate on this relevant example, modern radar Doppler systems employ frequency-hopping techniques to resist jamming. To still be effective, the jammer should then be able to identify the hostile radar frequency in a dynamic manner, and tune its frequency accordingly. The time over which the jammer is ineffective directly depends on the latency time of the IFM system employed. As a result, achieving latency times in the sub-100s ps range may be crucial in these applications, so that to minimize the time the jammer is unable to transmit at the correct frequency.

Owing to its low-power requirements, and the extreme compactness of the PS-WBG employed, the system presented here has a solid potential to be fully integrated on a single chip, including modulator and detectors. In turn, this may avoid the need for optical amplification, ultimately creating a fully integrated RF-photonic IFM system on-a-chip with ultralow latency, improved performance (for example, reduced frequency uncertainty) and relatively low cost. In particular, our choice for SOI technology is attractive because it leverages the maturity of the CMOS fabrication process to realize high-yield and low-cost electronic–photonic integrated circuits^17^,^23^. In addition, for this specific application, we believe that a crucial advantage of SOI compared with a III–V platform^24^ is the very high compactness and the possibility to monolithically integrate the RF and digital electronics together with the active/passive photonics components, without the need of hybrid integration in a package^23^,^25^,^26^, potentially leaving the laser as the only external component, with important benefits in terms of performance, yield and unit cost^17^,^27^.

Hence, to conclude, we believe the work reported here opens a very solid path towards the creation of compact RF frequency identification systems-on-a-chip that do not require any fast instrumentation, with direct applications to biomedical instrumentation, communications, defence, radio astronomy and more.

## Methods

### Grating design and layout

A Bragg grating with an effective index modulation *δn*≈0.042 was created by introducing 100-nm sidewall corrugations along the nanowire, which are visible in the scanning electron microscope image^17^ in Fig. 1b. The period is chosen to be 325 nm. A single phase-shift section with a length of 325 nm is included in the middle of the grating structure; 100 grating periods are fabricated on each side of the phase shift (Fig. 1a). The effective index modulation indicates the variation of the effective index of the corrugated waveguide forming the PS-WBG across the length of the grating. Wide sections will have an effective index (*n*+*δn*) and narrow sections (*n*−*δn*), where *n* is the effective index of the uncorrugated waveguide. The relationship between the effective index modulation and corrugation width of the waveguide has been extracted from experimental results on gratings with different corrugation widths, previously fabricated with the same 193-nm lithographic process^17^, and shows linear proportionality. The grating has been previously used for several signal-processing applications, including a fractional Hilbert transformer for terahertz-bandwidth optical pulses^18^.

### Test sequences in the dynamic experiments

Concerning the first generated dynamic temporal pattern, each sequence consists of a series of nine consecutive RF sinusoidal bursts, each with the duration of 167.225 ns, followed by a pause of the same duration. The sequence has been programmed in the AWG and reproduced with an internal clock frequency of 6 GSa s^−1^. As visible in Fig. 5a, the frequency hops in the following order: 0.6, 1.5, 3, 0.3, 2.4, 1.5, 0.6, 1.5 and 3 GHz. The total duration of one burst is 1.5 μs. The spectrogram (instantaneous frequency versus time) of the generated input signal is shown in Fig. 5e. The analogue output of the AWG is split into equal parts using a 3-dB RF splitter (Agilent 11667B, 0–26.5 GHz); one channel is fed directly to a 28-GHz real-time oscilloscope (Agilent DSO-X 92804A) and is displayed in Fig. 5a, and the other enters the IFM system in Fig. 3. For generation of the higher-frequency dynamic pattern, the internal clock of the AWG was set to its maximum (12 GSa s^−1^) and using the interleave function, it was possible to generate a sinusoidal burst up to 12 GHz (Fig. 5f–j).

### Data availability

The data that support the findings of this study are available from the corresponding author upon request.

## Additional information

**How to cite this article**: Burla, M. *et al*. Wideband dynamic microwave frequency identification system using a low-power ultracompact silicon photonic chip. *Nat. Commun.* **7,** 13004 doi: 10.1038/ncomms13004 (2016).

## Supplementary Material

Supplementary Information Supplementary Figures 1-7, Supplementary Notes 1-5 and Supplementary References.
