# Supplementary material for: Wideband dynamic microwave frequency identification system using a low-power ultracompact silicon photonic chip
Source: Nat Commun. 2016 Sep 30;7:13004. doi: 10.1038/ncomms13004 (PMC5427516; doi:10.1038/ncomms13004)
Supplement: Supplementary Information — Supplementary Figures 1-7, Supplementary Notes 1-5 and Supplementary References. [file ncomms13004-s1.pdf]

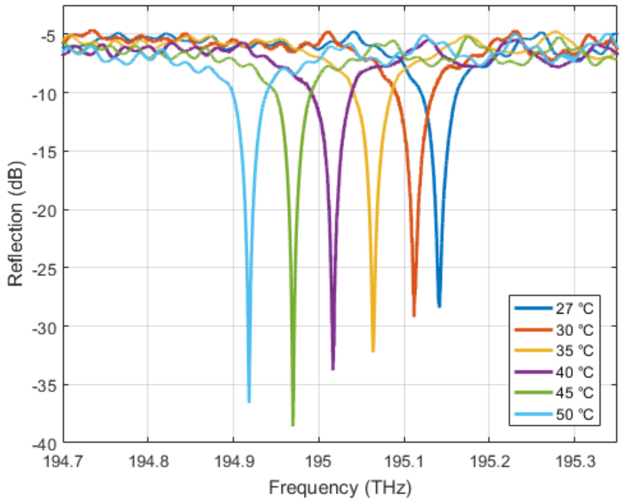

Supplementary Figure 1 | PS-WBG spectral response at different temperatures. Note how the frequency shifts with temperature, at a rate of approx. 9.44 GHz/°C.

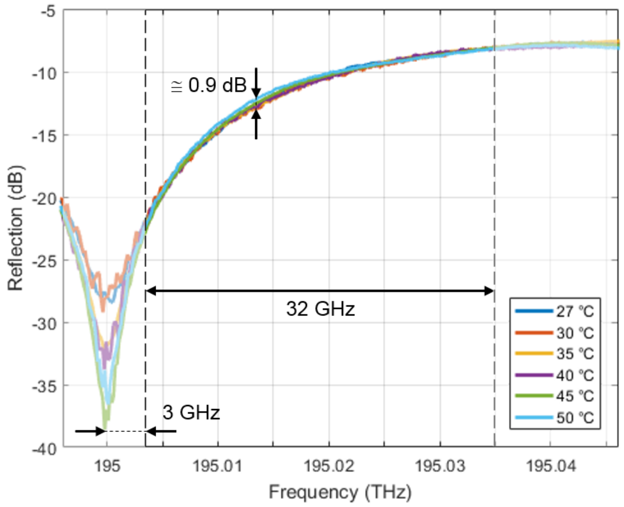

Supplementary Figure 2 | PS-WBG responses versus temperature. The shape of the PS-WBG response over the 32 GHz region of interest does not appear to vary sensibly with temperature.

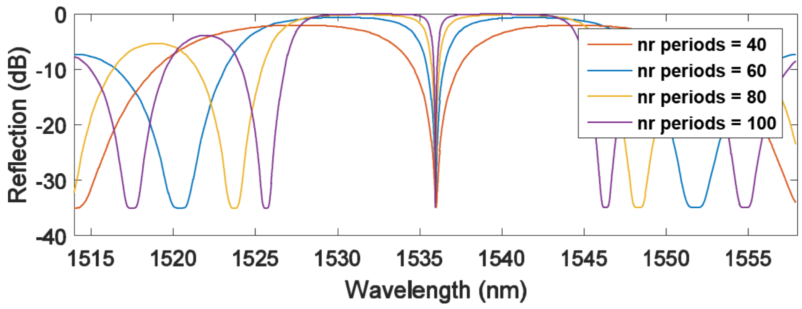

Supplementary Figure 3 | Simulated reflection spectra of PS-WBG, for different number of periods. Simulation obtained with transfer matrix method, grating period of 325 nm, effective index modulation δn = 0.042.

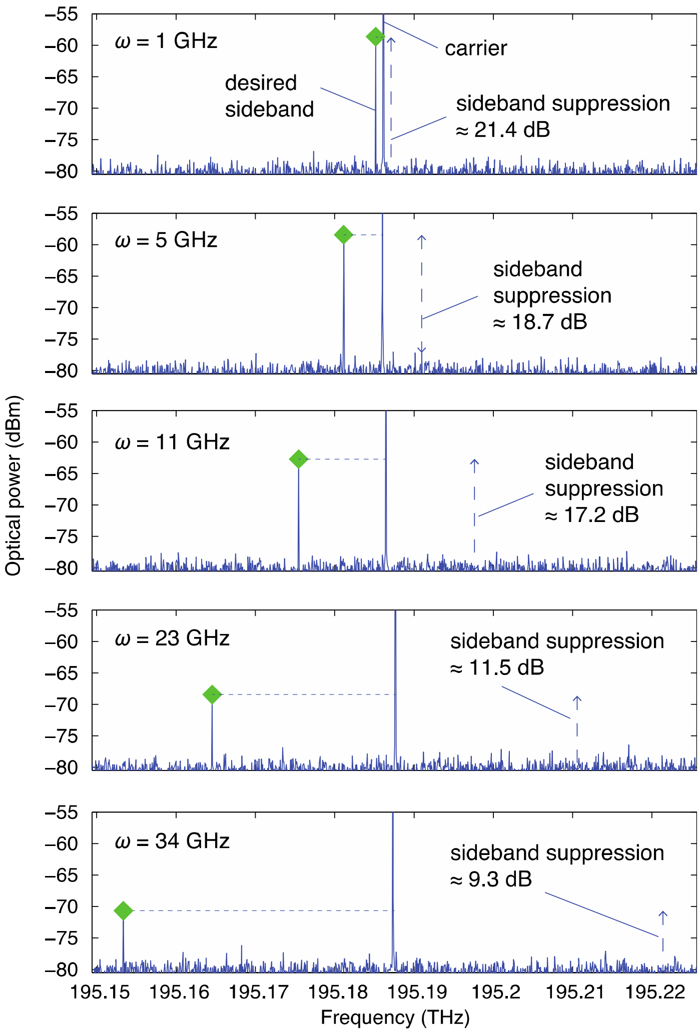

Supplementary Figure 4 | OSSB+C spectra. Optical single sideband spectra at different RF frequencies (ω).

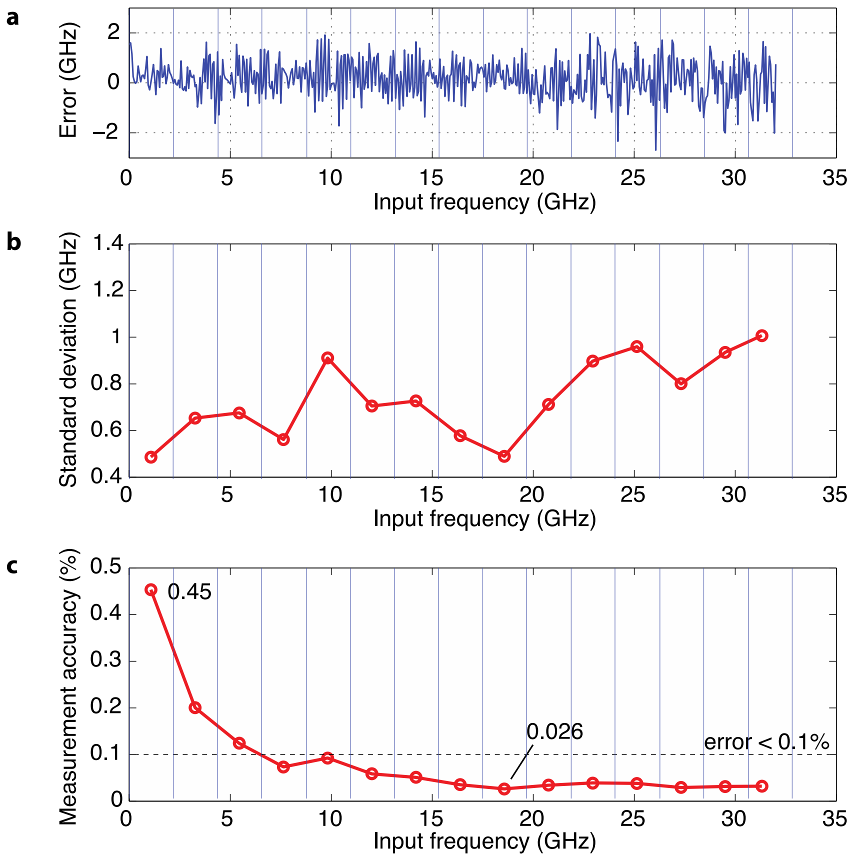

Supplementary Figure 5 | Error analysis. a, estimation error; **b**, standard deviation; **c**, measurement accuracy.

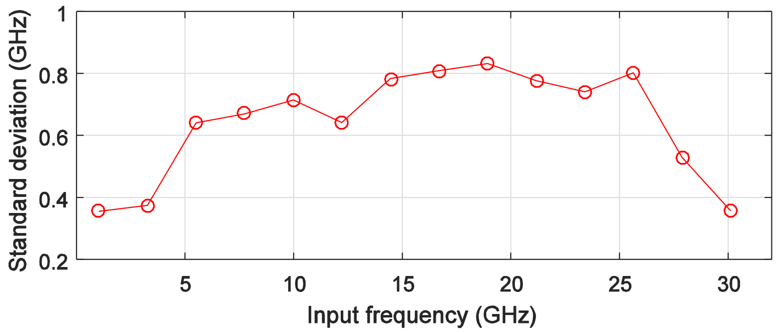

Supplementary Figure 6 | Theoretically estimated standard deviation of the frequency error. The average standard deviation over the complete range equals approximately 645 MHz (approx. 14.5% difference from the measured value of 755 MHz).

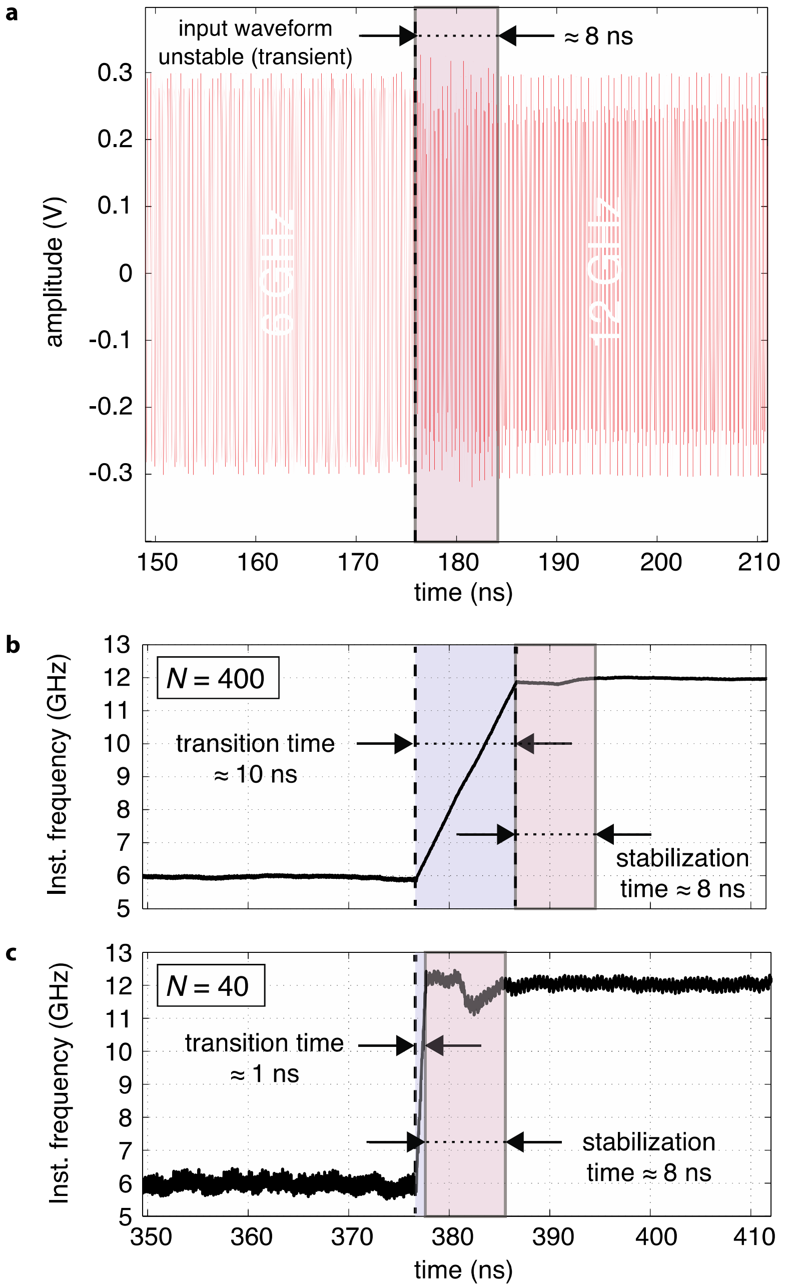

Supplementary Figure 7 | Transient response. a, Temporal detail of the input signal at the frequency transition from 6 GHz to 12 GHz; an 8-ns transient amplitude fluctuation is observed. **b**, Instantaneous dynamic frequency estimation obtained with $N = 400$ and (**c**) $N = 40$. Transition time reduces by reducing the value of N. An 8-ns transient effect is visible in the output signal in both cases due to the input amplitude fluctuations.

Supplementary Note 1: Temperature-dependence of the PS-WBG response

This section reports experimental characterization of the PS-WBG used in the IFM experiment with respect to temperature variations.

The optical response was measured at different temperatures (27, 30, 35, 40, 45 and 50 degrees Celsius) using the OVA. We limit our study to the reflection response because the transmission response shows complementarity, as shown in Fig. 2. The measured optical responses for different temperatures are shown in Supplementary Fig. 1.

As expected, the main effect of temperature variations on the PS-WBG is to shift the notch frequency in the reflection response. The central wavelength of the reflection notch changes sensibly with temperature, with a rate of approximately 9.44 GHz/°C. The resonance depth appears to vary of approximately 10 dB over the temperature span 27 – 50°C. Nonetheless, the shape of the response in correspondence of the 32 GHz-wide spectral region of interest (i.e. the transition band at the right of the notch, from 3 GHz to about 35 GHz from the notch, as shown in Fig. 2b, d) shows a limited variation and, most importantly, does not seem to be affected by drifts of the etalon fringes. For a clearer comparison, we numerically added a different frequency offset to each of the responses in Supplementary Fig. 1 so as to make the notches overlap at the same frequency (195 THz). The result is shown in Supplementary Fig. 2.

Therefore, we believe that the system could be correctly operated at different temperatures, by simply adjusting the laser wavelength according to the resonance of the PS-WBG filter and recalibrating the ACF response for the chosen temperature of

operation. This let us conclude that the etalon fringes should not be a major issue and should not impede operation at different temperatures. In addition, we envision that in applications the fibers would be fixed (e.g. glued) to the chip, as for example is being done with optical gyroscopes [1] or in commercial silicon transceivers [2]. This is expected to further reducing the effect of the etalon fringes over time and temperature.

Supplementary Note 2: Phase-shifted WBG spectra for different grating lengths

In this section we report the simulated optical reflection response of uniform phase-shifted waveguide Bragg gratings (PS-WBGs) with different number of periods (Supplementary Fig. 3). Simulations are performed using the transfer-matrix method (TMM) [3]. The period length is fixed to Λ = 325 nm. The effective index modulation is δn = 0.042. The effective index modulation can be related to the geometrical width corrugation of the waveguide [4].

Note that the PS-WBG shows a single notch over the complete reflection band. Reducing the number of periods increases the spectral width of the notch, and broadens the overall grating reflection band. The suppression in the notch does not degrade when reducing the number of periods of the grating.

Supplementary Note 3: Optical Single Sideband Spectra

A sideband ratio of 9.3 dB or higher is achieved over the complete bandwidth of operation by accurately biasing the DP-MZM. This is confirmed by the OSSB+C optical spectra (Supplementary Fig. 4), measured at the DP-MZM output using a ultra-high resolution optical spectrum analyzer (Apex Technologies AP2023B), for RF input tones of different frequency ω.

Supplementary Note 4: Frequency estimation error analysis

Photonic IFM systems usually show a tradeoff between frequency range and standard deviation. Therefore, a meaningful figure of merit is the ratio between the RF operating bandwidth and the standard deviation (BSD) offered by the IFM system:

$$\mathrm{BSD} = \frac{\mathrm{BW_{RF}}}{\sigma_{\mathrm{RF}}}. \tag{1}$$

The BSD in Supplementary Equation (1) is approximately equal to 42 for the system demonstrated here, which is comparable with the values obtained using other previously reported on-chip IFM systems (e.g., ≈37 for [5] over a bandwidth limited to ≈4 GHz).

More commonly in the published literature, authors report the percentage measurement accuracy, defined as the ratio between the standard deviation and the frequency range of interest:

$$\text{measurement accuracy, } \% = \frac{\sigma_{\mathrm{RF}}}{\mathrm{BW_{RF}}}. \tag{2}$$

Supplementary Fig. 5 provides a deeper analysis of the measurement error shown in Fig. 4. In order to estimate how the error varies with respect to the measurement frequency, we divide the frequency range of operation between 10 MHz and 32 GHz in 15 equal intervals. If we calculate the standard deviation of the error in each of these intervals, we observe that this quantity tends to degrade with frequency, reaching a maximum of approximately 1 GHz for the interval around 32 GHz, Supplementary Fig. 5**b**. However, if we now calculate the measurement accuracy in each interval, we observe an improvement with frequency, starting from 0.45% below 2 GHz and reaching a value of 0.026% around 18 GHz. This value is comparable with photonic IFM systems with state-of-the-art performance, that show record

measurement accuracy as low as 0.016% but use a relatively more complex setup, including 4 different lasers and 2.96 km of highly non-linear optical fiber (HNLF) [6]. It is important to note that the standard deviation of the error shown in Supplementary Fig. 5**b** seems to be nearly constant over RF frequency, despite both transmit and receive power noise increase with RF frequency, which could be counter-intuitive. This can be explained by considering that the ratio between the TX and RX responses gives a highly nonlinear ACF, which shows a higher slope at high RF frequency compared to low RF frequencies. This translates into the fact that the higher noise at high RF frequency is compensated by the higher slope (i.e. lower frequency sensitivity) of the ACF. Therefore, the same power error will induce a smaller frequency deviation at high frequency than at lower frequency. As a consequence, even if a smaller SNR is observed at higher RF frequencies, this induces a similar frequency estimation error.

Theoretical measurement error. Based on the optical losses and noise figure of the components in the IFM system, we performed a system simulation to estimate the theoretical frequency measurement error and compared it with the experimental results. The different sources of noise have been considered. For our photonic link, the total noise power can be calculated as

$$p_n = (1+g)p_{\mathrm{th}} + \frac{1}{4}p_{\mathrm{shot}} + \frac{1}{4}p_{\mathrm{rin}} + \frac{1}{4}p_{\mathrm{sys}}. \tag{3}$$

Where p_{th} is the thermal noise power originating from the modulator and from the photodetector matching resistors, $p_{\mathrm{rin}}$is the relative intensity noise (RIN) from the laser, and $p_{\mathrm{shot}}$is shot noise due to the random arrival of photons at the photodetectors. These are the noise contribution in a basic photonic link [7][8], composed by a modulator, a fiber link and a photodetector. The ¼ terms originate from the lossy impedance matching condition employed in the experiments. For our

system, we had to add additional terms, i.e. the noise originating from the optical system comprising the EDFAs, the polarization controller, and the photonic chip. We lumped these together in the term p_{sys}. The overall noise factor F_{sys} of the photonic system is related to the noise factor of the individual components through Friis' formula [9][10]:

$$F_{\text{sys}} = F_{\text{EDFA1}} + \frac{F_{\text{PC}} - 1}{g_{\text{EDFA1}}} + \frac{F_{\text{chip}} - 1}{g_{\text{EDFA1}} \cdot g_{\text{PC}}} + \frac{F_{\text{EDFA2}} - 1}{g_{\text{EDFA1}} \cdot g_{\text{PC}} \cdot g_{\text{chip}}} \tag{4}$$

where F_{EDFA1}, F_{EDFA2}, F_{PC}, F_{chip} are the noise factors of the first and second EDFAs, of the polarization controller and of the photonic chip, respectively, and g_{EDFA1}, g_{EDFA2}, g_{PC}, g_{chip} are the corresponding gain terms. From the definition of noise figure [10], the noise power from the photonic system is:

$$p_{\text{sys}} = (F_{\text{sys}} - 1) g_{\text{sys}} kTB. \tag{5}$$

Using this approach, we estimated the noise power spectral density of the transmission link and of the reflection link separately. Then, employing the ACF in Fig. 2**f**, we could estimate the theoretical standard deviation of the measurement error of the complete system.

The estimated standard deviation of the frequency error over the complete frequency range is reported in Supplementary Fig. 6.

Analysing the standard deviation versus frequency, it appears to be relatively constant across RF frequency, in agreement with the experimental result displayed in Supplementary Fig. 5**b**.

The average value of the standard deviation of the frequency error over the complete frequency range approximately equal to 645 MHz, versus the 755 MHz measured experimentally, corresponding to a difference of 14.5%. We attribute this discrepancy between the simulated and measured frequency error mainly to the uncertainties in

knowledge of the exact values of the system parameters. This simulation is based on the noise figure and gain values reported on the EDFA datasheet. These are typical values and may differ from the ones observed in our specific experimental conditions, explaining the slight deviation. In addition, the system noise equivalent bandwidth B used in the calculation of the noise terms depends on the shape of the filter characteristics [8], which is not exactly known. For simplicity, this was approximated to be equal to the nominal 3 dB bandwidth of the measurement instrumentation used.

Supplementary Note 5: Transient analysis

In this section, we discuss the transient response of the dynamic IFM system. In particular, we analyze the "locking time" or "settling time", intended here as the time needed by the system to reach and keep the correct frequency indication after the input signal undergoes an arbitrary frequency variation.

In order to characterize the transient response of the system, we analyze the behavior of the output signal $f_{\mathrm{inst}}(t)$ in proximity of the time instants at which the RF frequency of the input signal changes. Supplementary Fig. 7**a** shows a temporal detail of the input signal in Fig. 5**a**, in proximity of the transition from 6 GHz to 12 GHz of the waveform generated by the AWG. In Supplementary Fig. 7**b** we show the corresponding variation of the output signal $f_{\mathrm{inst}}(t)$. The axes of the two figures have been vertically aligned to remove the latency of the system.

The output reaches a stable and correct frequency indication after approximately 18 ns from the end of the 6 GHz burst. This time interval can be divided in a *transition time* of about 10 ns, during which the signal rises from 6 GHz to 12 GHz, and a *stabilization time* of approximately 8 ns. The duration of the transition time is in line with the time constant ΔT chosen for the moving average filter in equation (2). The

output fluctuations during the stabilization time, instead, are attributed to the fact that the input waveform shows a transient amplitude fluctuation after the signal changes its frequency from 6 to 12 GHz. Similar fluctuations are visible in Fig. 5**a** also after the RF signal changes its frequency from 2.4 GHz to 6 GHz. This effect is due to the limited RF bandwidth of the employed AWG.

With a settling time below 20 ns, the proposed system matches or surpasses the performance requirements of state-of-the-art IFM systems. For example, in a typical frequency hopping spread spectrum (FHSS) communication system, rates from tens up to several hundreds hops per second are practical, requiring frequency identification times in the order of hundreds of microseconds [11]. In electronic warfare applications, where stricter requirements are demanded, IFM systems should be able to identify pulses with duration in the order of 100 ns [12].

It is worth noting that the transition time is not limited by the photonic IFM system, but instead by the moving average filter employed to extract the signal power. To prove this statement, we reduced the number of samples employed in the moving average filter in Fig. 5 from $N = 400$ to $N = 40$, corresponding to a time constant reduction from 10 ns to 1 ns. The output signal obtained with this new filter is shown in Supplementary Fig. 7**c**. The transition time is now of the order of 1 ns only. Also in this case, after the signal rises from 6 to 12 GHz, further fluctuations can be seen; there is a stabilization time in the order of 8 ns, which again is due to the transient amplitude fluctuations in the input signal. The output waveform in Supplementary Fig. 7**c** shows higher uncertainty compared to Supplementary Fig. 7**b**. This is due to the broader passband of the low-pass filter obtained with $N = 40$, which causes a more significant residual component of the original radiofrequency signal to be observed at the output.

This discussion shows that a tradeoff exists between the minimum frequency that can be detected, and how fast the IFM can track rapid frequency variations of the input signal. Therefore the number of points *N* in the moving average filter should be tailored according to the expected frequency range of the unknown microwave input signal and the required tracking speed. A smaller number of points can be chosen for identifying higher frequency input signals, reducing the system response time, as we have shown in our example.

We emphasize again that a relatively slow electronic comparator, with 3-dB bandwidth of a few hundreds MHz, would be sufficient to correctly identify the reported tens of GHz-level microwave frequencies, including the nanosecond-scale dynamic transitions between them [13].

Supplementary References

[1] Guillén-Torres, M. Á. *Feasibility of optical gyroscopic sensors in silicon-on-insulator technology*. PhD thesis, University of British Columbia (2015).

[2] Fischer-Hirchert, U. H. P. *Photonic Packaging Sourcebook*. Springer, 2015.

[3] Born, M., Wolf, E. *Principles of optics: electromagnetic theory of propagation, interference and diffraction of light*. Oxford, Pergamon Press, 1964.

[4] Chrostowski, L. and Hochberg, M. *Silicon Photonics Design* (Cambridge University Press, Cambridge, 2015).

[5] Marpaung, D. On-Chip Photonic-Assisted Instantaneous Microwave Frequency Measurement System. *IEEE Photon. Technol. Lett.* **25,** 837–840 (2013).

[6] Emami, H., Ashourian, M. and Ebnali-Heidari, M. Dynamically Reconfigurable All Optical Frequency Measurement System. *Journal of Lightw. Technol.* **32,** 4194–4200 (2014).

[7] Cox C.H., *Analog Optical Links: Theory and Practice.* (Cambridge University Press,

Cambridge, 2004).

[8] Marpaung D.A.I. *High Dynamic Range Analog Photonic Links - Design and Implementation.* Ph.D. thesis, University of Twente, Enschede, The Netherlands, 2009.

[9] Friis, H.T. *Noise Figures of Radio Receivers*, Proc. of the IRE, July, 1944, pp. 419-422.

[10] Agilent application note 57-1, "*Fundamentals of RF and Microwave Noise Figure Measurements*". Online. Available: http://cp.literature.agilent.com/litweb/pdf/5952-8255E.pdf

[11] Texas Instrument application note AN014, "Frequency Hopping Systems". Online. Available: http://www.ti.com/lit/an/swra077/swra077.pdf

[12] Takeuchi, Y. Instantaneous Frequency Measurement System. Nov. 9, 1976. U.S. Patent 3,992,365.

[13] National Instruments Knowledge Base. Online. Available: http://digital.ni.com/public.nsf/allkb/ A4530BE56775FB2C86257282005EAB58
